# A global overview of the use of cone beam computed tomography in dentistry: a bibliometric review focusing on paediatric patients

**DOI:** 10.12688/f1000research.157349.1

**Published:** 2024-11-05

**Authors:** Danielle Cristina Alves Rigo, Aurélio de Oliveira Rocha, Lucas Menezes dos Anjos, Pablo Silveira Santos, Isabela Ramos, Michely Cristina Goebel, Julia Maldonado Garcia, Gabriela Beatriz Rigo Wietzkoski, Carla Miranda Santana, Mariane Cardoso

**Affiliations:** 1 Department of Dentistry, Postgraduate Program of Dentistry, Federal University of Santa Catarina, Florianópolis, State of Santa Catarina, 88040-370, Brazil; 2 Department of Medicine, Graduate Program of Medicine, São Lucas University Centre (UNISL), Porto Velho, Rondônia, Brazil

**Keywords:** Cone-Beam Computed Tomography, CBCT, Paediatric, Dentistry, Bibliometric Analysis

## Abstract

**Background:**

Cone Beam Computed Tomography (CBCT) has improved diagnosis and treatment planning in paediatric dentistry, but no bibliometric studies have examined the research landscape. This study provides an overview of CBCT in paediatric dentistry.

**Methods:**

A bibliometric review was conducted using articles from the Web of Science database up to February 2024. Conference papers and editorials were excluded. Data extracted included citation counts, publication dates, journals, impact factors, study designs, topics, geographical and institutional affiliations, authors, and keywords. Collaborative networks were visualised using VOSviewer, and Spearman’s correlation assessed the relationship between citation counts and other variables.

**Results:**

The review analysed 517 articles, with the most cited receiving 557 citations. Publication dates ranged from 2005 to 2024, with a peak in 2023. Observational studies were the most common, particularly on maxillary expansion. The American Journal of Orthodontics and Dentofacial Orthopedics was the most cited journal, and the USA was a major contributor. Jacobs R authored the most articles (n=19), and the University of Alberta led in institutional output. Spearman’s correlation showed a weak positive correlation between citation count and journal impact factor (rho=0.272, p<0.001) and a strong negative correlation with publication year (rho=-0.762, p<0.001).

**Conclusions:**

This bibliometric review provides an overview of the use of CBCT in paediatric dentistry, particularly in maxillary expansion. The findings suggest that more specific imaging protocols may improve safety and clinical outcomes, and that further investigation of long-term outcomes may provide valuable insights.

## Introduction

Cone beam computed tomography (CBCT) represents a diagnostic imaging approach using x-radiation to reproduce a specific section of the human body in any of the three planes of space. ^1^ In contrast to conventional radiographs, which project all the structures traversed by X-rays in a single plane, CBCT highlights structural relationships in depth, presenting images in “slices”. ^2^ Unlike multi-slice computed tomography (MSCT), CBCT uses an X-ray source composed of a fixed low-energy anode, similar to those used in dental panoramic tomography. ^3^ Despite having a lower incidence of radiation than MSCT, CBCT generates high-resolution images and minimizes the likelihood of metallic artefacts. In addition, it offers the ability to generate all two-dimensional images as required. ^4, 5^

Although CBCT is a growing imaging technology with several dentomaxillofacial applications, its use in paediatric dental patients remains a sensitive point, mainly due to the radiation doses involved. ^6^ Specific recommendations aimed at guiding the justified indication of the use of CBCT in paediatric dental patients were discussed as part of the European project DIMITRIA (Paediatric dentomaxillofacial imaging: an investigation into low-dose radiation-induced risks), which focused on optimizing the doses applied to children. ^7^ Despite the need for a cautious approach, particularly for the investigation and appropriate treatment of orofacial and jawbone pathologies, as well as specific situations such as dental trauma, orofacial clefts and supernumerary teeth, the use of CBCT is justified. ^4^ Given this scenario, the use of CBCT in paediatric dental patients has emerged as a crucial imaging exam to help professionals diagnose and prognosticate numerous oral conditions in children. ^5, 8, 9^

Despite the widespread use of CBCT in paediatric dental care, there is a lack of comprehensive bibliometric analyses that examine the trends, geographical distribution, and scientific collaborations in this area. Many studies have focused on the technical aspects and clinical applications of CBCT, ^1, 3– 5, 7– 9^ but no studies have been found to date that have systematically mapped the research landscape to identify gaps in knowledge and areas that need further investigation. Furthermore, concerns about radiation exposure in children underscore the importance of establishing clearer protocols and guidelines for its safe application. Existing studies tend to concentrate on specific clinical outcomes, leaving a broader understanding of CBCT’s impact on paediatric dental care largely unexplored.

Bibliometric studies map existing concepts, indicating the main trends in scientific research and the main dental methods and tools used. ^10, 11^ These analyses allow researchers to identify patterns, assess influential indicators, recognise leading authors, evaluate prominent journals, and highlight countries with the most significant research output. Additionally, bibliometric analysis enables the visualisation of collaborative networks between researchers. ^12^ Although bibliometric studies have been conducted in several fields of dentistry, ^13– 16^ no studies to date have specifically focused on the use of CBCT in paediatric dentistry. In light of this gap, the aim of this study was to conduct a bibliometric analysis to provide a comprehensive panorama on the scientific landscape of CBCT on paediatric dentistry and to guide future clinical and research developments.

## Methods

This study complies with the Guideline for Reporting Bibliometric Reviews of Biomedical Literature (BIBLIO) ^17^ and follows a rigorous standard of data collection and analysis. ^17, 18^

A literature review was conducted to identify studies using CBCT in paediatric dental patients. The search covered publications up to February 2024, with no restrictions on language or publication year. The bibliographic search was conducted in the Web of Science - Core Collection (WoSCC) database, using the following search strategy: [TS=(“Cone-Beam Computed Tomography” OR “Cone Beam Computed Tomography” OR CBCT OR “Computed Tomography, Cone-Beam” OR “Cone Beam Computed Tomography” OR “CT Scan, Cone-Beam” OR “CT Scan, Cone Beam” OR “CT Scans, Cone-Beam” OR “Cone-Beam CT Scan” OR “Cone-Beam CT Scans” OR “Scan, Cone-Beam CT” OR “Scans, Cone-Beam CT” OR “Tomography, Cone-Beam Computed” OR “Tomography, Cone Beam Computed” OR “CAT Scan, Cone-Beam” OR “CAT Scan, Cone Beam” OR “CAT Scans, Cone-Beam” OR “Cone-Beam CAT Scan” OR “Cone-Beam CAT Scans” OR “Scan, Cone-Beam CAT” OR “Scans, Cone-Beam CAT” OR “Cone-Beam Computer-Assisted Tomography” OR “Computer-Assisted Tomography, Cone-Beam” OR “Cone Beam Computer Assisted Tomography” OR “Tomography, Cone-Beam Computer-Assisted” OR “Cone-Beam Computerized Tomography” OR “Computerized Tomography, Cone-Beam” OR “Cone Beam Computerized Tomography” OR “Tomography, Cone-Beam Computerized” OR “Cone-Beam CT” OR “CT, Cone-Beam” OR “Cone Beam CT”) AND ((“Paediatric Dentistry” OR “Dentistry, Paediatric” OR “Paediatric Dentistry” OR “Dentistry, Paediatric”) OR (child* OR schoolchild* OR “school child*” OR adolescen* OR juvenil*) OR (“tooth, deciduous” OR “deciduous tooth” OR “deciduous teeth” OR “milk teeth” OR “milk tooth” OR “primary teeth” OR “primary tooth” OR “primary dentition”))]. Two researchers independently conducted the study selection process, and a third author was consulted in the event of disagreements. Studies were only included when all the reviewers reached a consensus. Studies that were not research articles (such as conferences or editorials) and did not use CBCT in paediatric dental patients were excluded.

For each article, data was collected such as number and density of citations (number of citations per year), year of publication, journal impact factor (JIF), study design, theme (main objective of the study), country and continent, institution (based on the corresponding author), authors and keywords. The study designs were categorized as systematic review, interventional, observational, case report or series, literature review and laboratory study. Considering the theme, the studies were grouped considering the central outcomes of each study. Themes that were reported in only one study were grouped under “other”.

The selected articles were entered into the VOS Viewer software (version 1.6.18) ( https://www.vosviewer.com) to create visual representations that show collaboration between authors and keywords. Words associated with the most prominent foci indicate greater frequency, while words of the same color and linked together form networks, highlighting more intense collaboration between studies. The correlation between the number of citations, the year of publication and the journal’s impact factor was explored using SPSS for Windows statistical software (SPSS, version 24.0; IBM, Armonk USA Corp). The Kolmogorov-Smirnov test was used to verify the normality of the data distribution, and Spearman’s correlation test was used due to the non-normal distribution. As an alternative, Microsoft Office Excel can be used to carry out these statistical calculations (normality test and correlation), with the relevant database provided in the Data Availability section. ^19^

## Results

The literature search identified a total of 1,147 records. After the selection process, 517 articles were included for bibliometric analysis. Of the articles not included, two were editorials, three were from conferences, and the remaining (n=625) were excluded because they did not address the objective of the study. ^19^

The selected articles accumulated a total of 7,538 WoSCC citations. Of these, 896 were self-citations (11.53%). The average number of citations per year was 396.7. The most cited article on WoSCC was “Effective dose of dental CBCT-a meta-analysis of published data and additional data for nine CBCT units”, with 257 citations, published in Dentomaxillofacial Radiology. ^20^ Spearman’s correlation revealed a weak positive correlation between the number of citations and the journal’s impact factor (rho=0,272; p<0.001) and a strong negative correlation between the number of citations and the year of publication (rho=-0.762; p<0.001).

**Figure 1.**
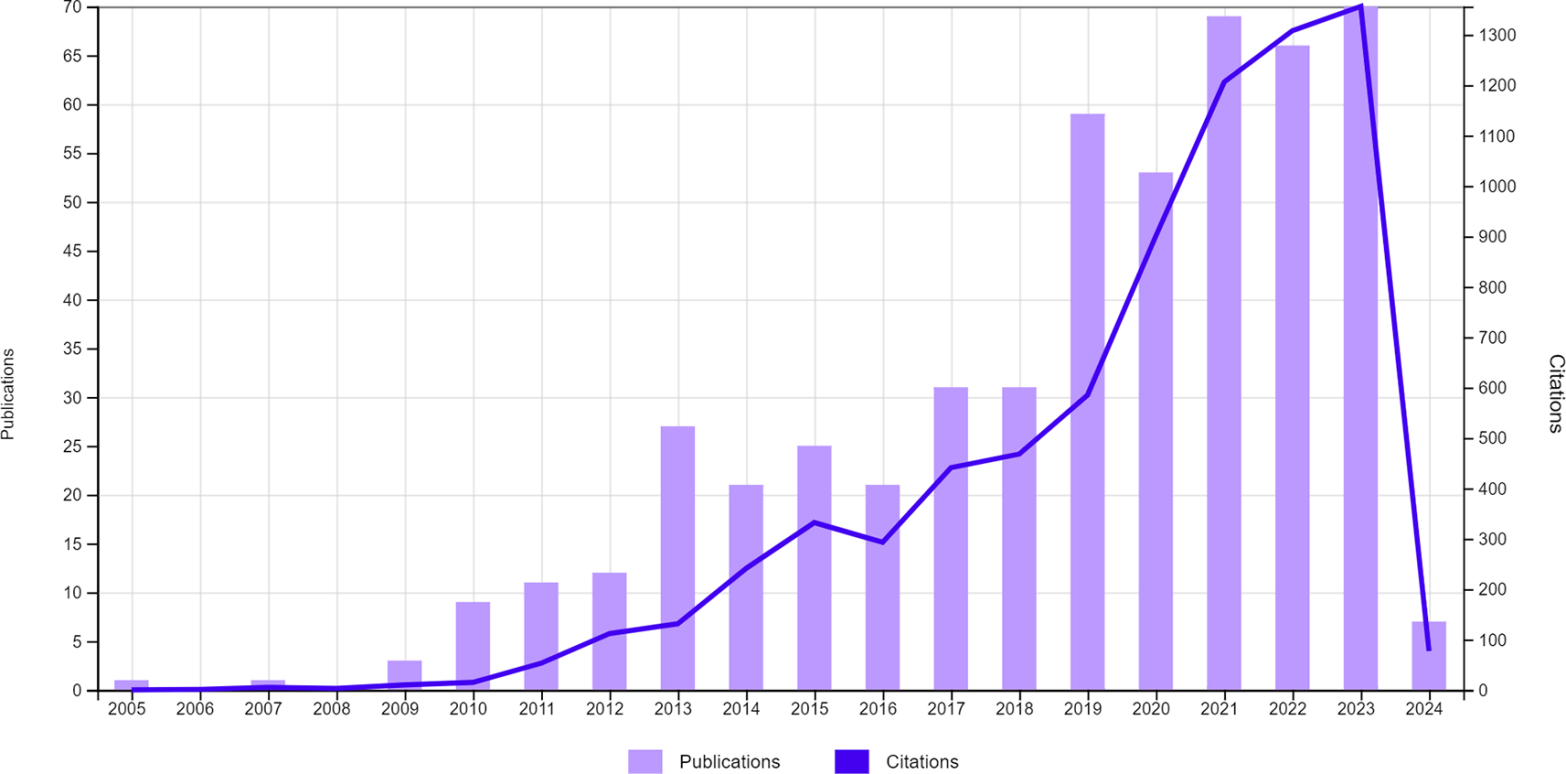
Distribution of the number of publications over the years.

The articles were published between 2005 and 2024, spanning 19 years. The earliest publications date back to 2005, ^7^ while the most recent, up to February 2024, total two. ^21, 22^ The year with the highest number of publications was 2023, with 71 articles indicating a recent and significant interest in research related to this topic. The distribution of the number of publications over the years can be seen in Figure 1.

The most frequent journals that published articles on the use of CBCT in paediatric dental patients are shown in Table 1. The American Journal of Orthodontics and Dentofacial Orthopedics (n=69) was the most prominent journal, followed by Angle Orthodontist (n=30) and Dentomaxillofacial Radiology (n=23). According to Journal Citation Reports, the journals with the highest IF in 2022 linked to this study were the Journal of Dental Research (IF 7.7; 1 occurrence), European Radiology (IF 5.9; 2 occurrences) and the International Endodontic Journal (IF 5.0; 1 occurrence), all contributing one article each.

**Table 1.** Top 10 journals with the highest number of publications.

Source Title	Number of papers	Number of citations	Journal Impact factor
American Journal Of Orthodontics And Dentofacial Orthopedics	69	2.583	3.0
Angle Orthodontist	30	743	3.4
Dentomaxillofacial Radiology	23	591	3.3
BMC Oral Health	21	92	2.9
Clinical Oral Investigations	17	233	3.4
Orthodontics & Craniofacial Research	14	95	3.1
Journal of Craniofacial Surgery	13	89	0.9
European Journal of Paediatric Dentistry	11	123	3.6
International Journal of Paediatric Dentistry	11	114	3.8
Dental Traumatology	11	81	2.5

Most of the articles were observational studies (n=352), followed by laboratory studies (n=54), case reports or series (n=39), interventional studies (n=41), literature reviews (n=20) and systematic reviews (n=11). About the themes of the selected studies, the majority of studies used CBCT to plan and assess maxillary expansion (n=87), followed by aid in endodontic diagnosis and treatment (n=39) and to aid in the diagnosis and treatment of cleft lip and/or palate (n=38). Table 2 lists the different themes with their respective study designs and the year in which the articles were published.

**Table 2.** Characteristics of the studies in relation to the theme.

Thematic	Study design	Number of papers	Number of citations
**Maxillary expansion (n=87)**	Systematic review	1	2
	Intervention	28	862
	Observational	51	973
	Case report or series	3	8
	Literature review	2	105
	Laboratory study	2	1
**Aid in endodontic diagnosis and treatment (n=39)**	Intervention	2	10
	Observational	14	104
	Case report or series	1	0
	Literature review	2	44
	Laboratory study	20	211
**Aid in the diagnosis and treatment of cleft lip and/or palate (n=38)**	Systematic review	1	8
	Observational	36	305
	Case report	1	45
**Clinical conditions associated with the Angle classification (n=36)**	Systematic review	1	0
	Intervention	3	19
	Observational	30	226
	Case report or series	2	3
**Dental impaction (=36)**	Systematic review	1	4
	Intervention	3	85
	Observational	25	313
	Case report	7	12
**Facial anatomy (n=35)**	Systematic review	1	0
	Observational	30	865
	Literature review	1	1
	Laboratory study	3	13
**Diagnosis and treatment of bone lesions (n=31)**	Observational	22	109
	Case report or series	6	23
	Literature review	3	0
**CBCT in orthodontic treatment (n=30)**	Systematic review	2	65
	Observational	20	159
	Case report or series	3	45
	Laboratory	3	75
**Radiation dose assessment (n=29)**	Systematic review	1	17
	Intervention study	1	6
	Observational	8	297
	Literature review	3	340
	Laboratory study	17	359
**Dental anatomy (n=27)**	Observational	26	259
	Laboratory study	1	46
**Facial growth (n=23)**	Systematic review	2	42
	Observational	19	139
	Case report or series	1	3
	Literature review	1	3
**Trauma (n=14)**	Intervention	1	3
	Observational	5	30
	Case report or series	7	10
	Laboratory study	1	13
**Oral and facial changes associated with juvenile idiopathic arthritis (n=12)**	Observational	12	93
**Indications for CBCT (n=10)**	Observational	9	30
	Laboratory study	1	0
**Identification of cephalometric points (n=10)**	Observational	9	254
	Literature review	1	42
**Autotransplatantation (n=9)**	Observational	3	36
	Case report or series	5	54
	Laboratory study	1	8
**Aid in the diagnosis and treatment of temporomandibular dysfunction (n=7)**	Intervention	1	29
	Observational	5	113
	Case report or series	1	0
**Diagnosis and treatment of caries (n=4)**	Intervention	1	8
	Observational	2	32
	Laboratory study	1	8
**Oral and facial changes in patients with Down Syndrome (n=3)**	Observational	3	25
**Scientific and other status (n=33)**	Systematic review	2	40
	Observational	21	148
	Case report	2	10
	Literature review	5	174
	Laboratory study	3	53

**Figure 2.**
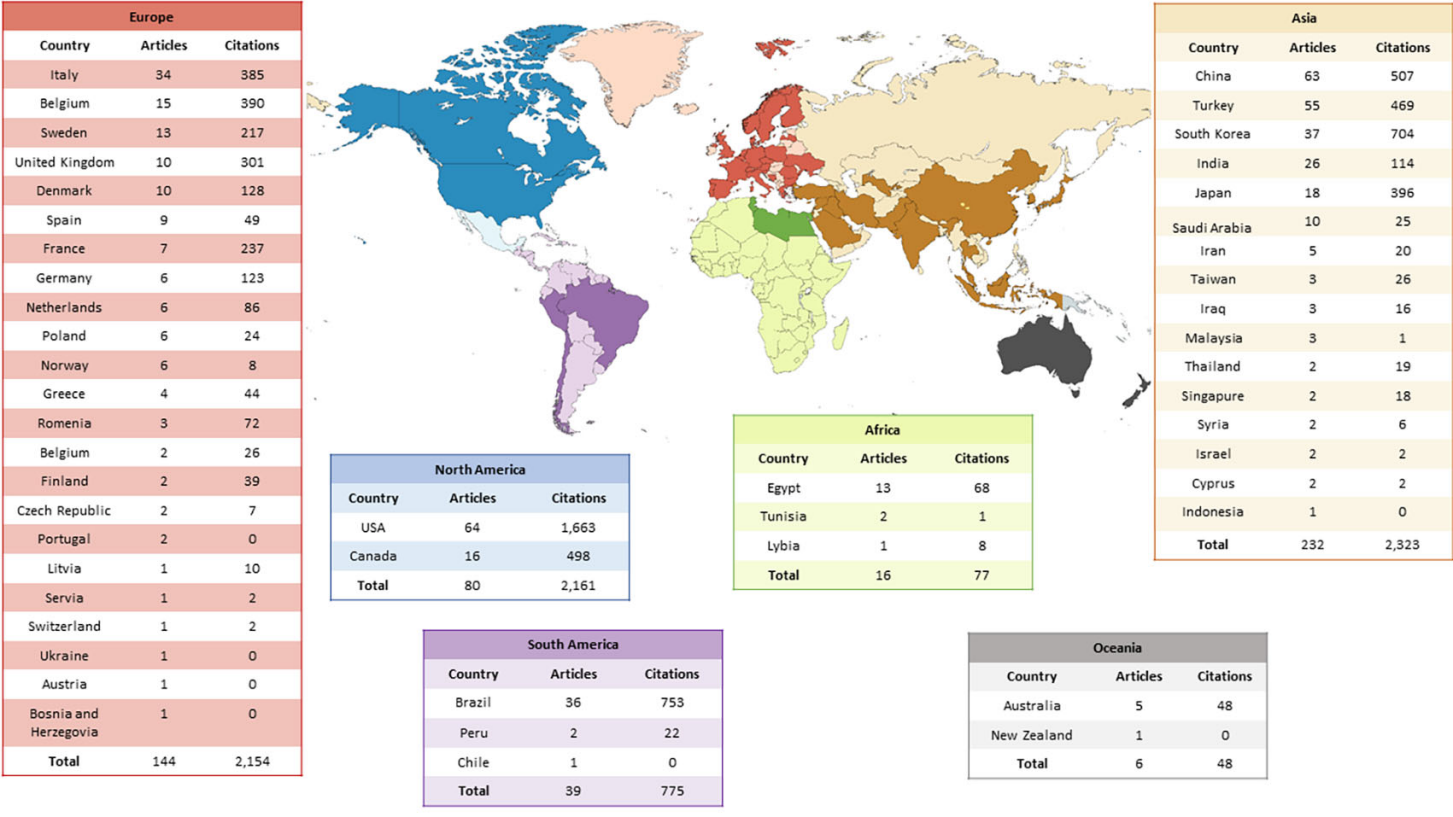
Worldwide distribution of the use of CBCT in paediatric dental patients.

A total of 52 countries contributed to the articles related to the use of CBCT in paediatric dental patients. Considering the number of publications per country, the most prevalent were the USA (n=64), China (n=63), Turkey (n=55), South Korea (n=37) and Brazil (n=36). Among the continents with the most articles, Asia stands out (n=232), followed by Europe (n=144) and North America (n=80). The worldwide distribution of publications can be seen in Figure 2.

Through the data contained in the corresponding authors of the selected studies, 301 different institutions were identified. The University of Alberta (Canada) was the institution with the largest number of documents (n=11). Table 3 shows the top 10 institutions.

**Table 3.** Main institutions associated with publications on the use of Cone Beam Computed Tomography in Paediatric dental patients.

Institution	Country	Number of papers	Number of citations
University of Alberta	Canada	11	438
Katholieke University Leuven	Belgium	9	280
Kagoshima University	Japan	9	24
Kyung Hee University	South Korea	9	216
Peking University	China	9	216
University of São Paulo	Brazil	8	62
Catholic University of Korea	South Korea	6	139
Erciyes University	Turkey	6	93
Pusan National University Hospital	South Korea	6	39
University of Pennylvania	USA	7	146

**Figure 3.**
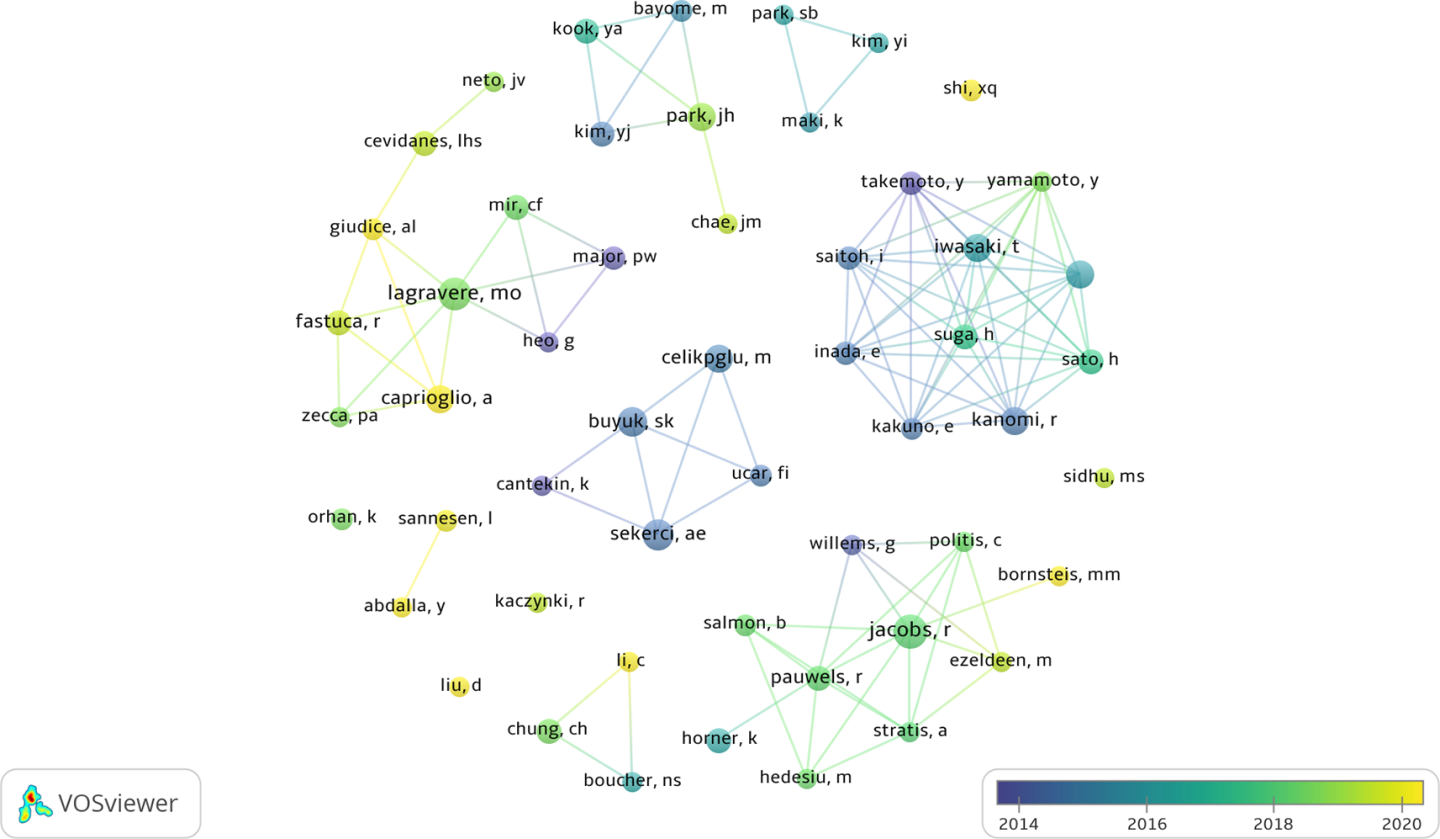
Description and collaboration between the main authors.

The authors with the highest number of articles were Jacobs R (n=19), Lagravere MO (n=16) and Sekerci AE (n=14). Figure 3 highlights the collaboration and the main groups of authors, while Table 4 shows the 10 authors with the highest number of publications.

**Table 4.** Top 10 authors with the most published articles.

Authors	Number of papers	Number of citations	Number of papers in WoS-CC	Number of citations in WoS-CC	H-Index
Jacobs R	19	679	603	18.527	71
Lagravere MO	16	449	90	2.224	26
Sekerci AE	14	198	72	1.062	19
Buyuk SK	12	142	29	449	14
Kanomi R	11	344	35	1.023	16
Iwasaki T	11	293	31	619	13
Celikpglu M	11	124	77	1.345	24
Yamasakie Y	10	284	88	988	15
Park JH	10	137	70	1.182	22
Caprioglio A	10	131	55	711	17

**Figure 4.**
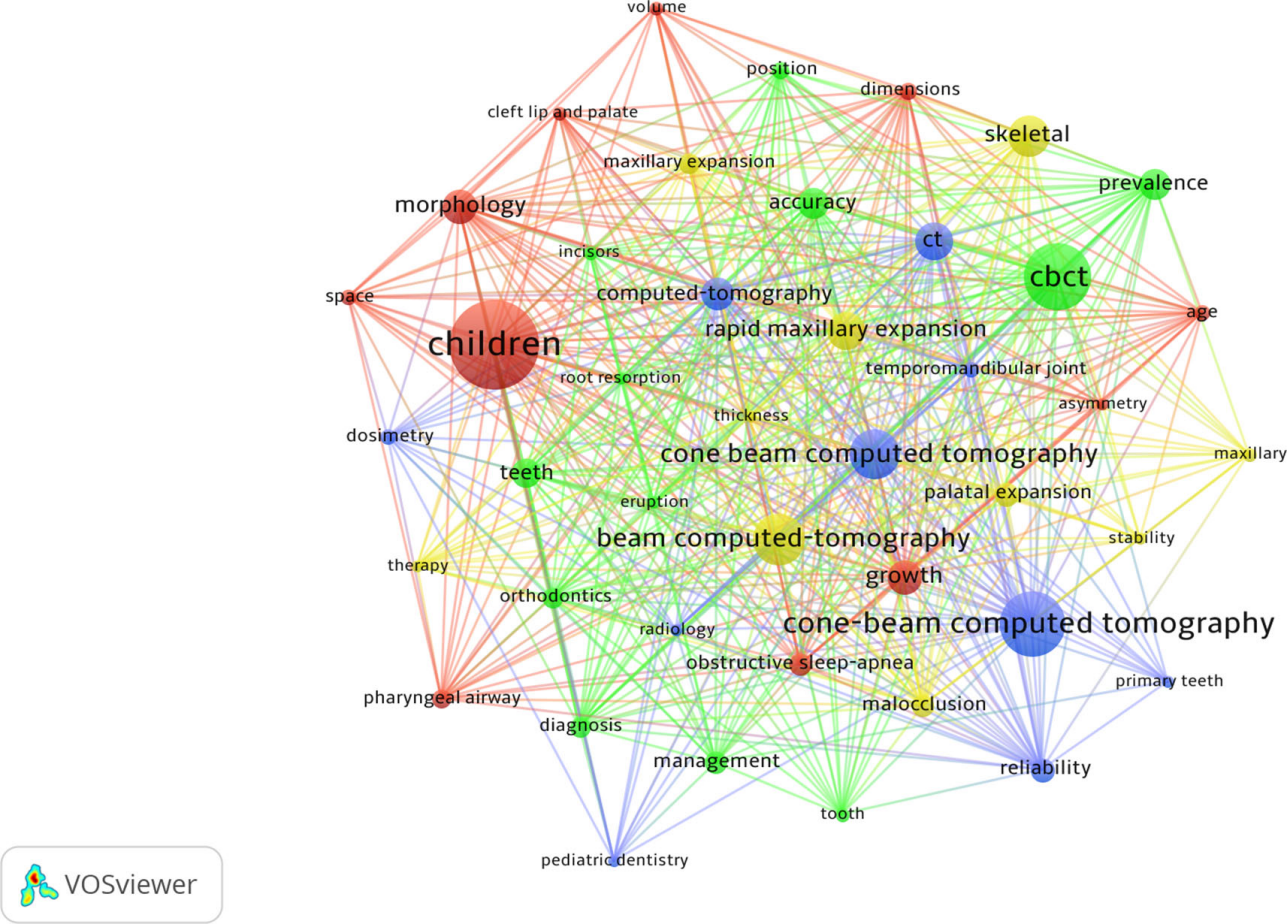
Description and collaboration between the main keywords.

A total of 1,841 keywords were identified. The most prevalent was “children” (n=118), followed by “CBCT” (n=85) and “cone-beam computed tomography” (n=83). Figure 4 shows the most prevalent keywords (5 or more occurrences) and the collaborative relationships between them.

## Discussion

In paediatric dental patients, CBCT plays a crucial role in diagnosing oral conditions such as dental fractures and bone lesions. ^4^ In addition, it serves as an essential tool in planning procedures such as maxillary expansion and correction of nasopalatine clefts. ^23^ This study identified the main applications of CBCT in paediatric dental patients, analysed the profile of publications related to this topic and discussed possible future trends for research in this field. The results revealed that the emphasis is predominantly on observational studies, concentrating on the diagnosis and planning of patients with maxillary atresia who require expansion procedures.

### Citation analysis

The most cited article, published in 2015, is a systematic review that aimed to analyse dose measurement and estimate the effective dose for performing CBCT with dental applications. ^20^ In this study, the authors indicated that the effective dose for performing CBCT in children ranges from 13 to 769 μSv for large or medium fields of view and 7 to 521 μSv for small FOV fields of view. The abundance of citations in this study highlights not only the importance of using CBCT in dental applications but also underscores the crucial need for accurate measurement of radiation exposure, especially in paediatric dental patients.

A cohort study carried out in the UK, involving approximately 175,000 individuals who were children when exposed to CT scans of the head, revealed that cumulative doses of around 50 mGy almost tripled the risk of leukaemia, while doses of around 60 mGy almost tripled the risk of brain cancer. ^24^ Similar results were observed in an Australian cohort of 10.9 million people aged between 0 and 19, showing a 24% increase in the incidence of cancers, including brain cancers and leukaemia, after exposure to CT scans. This incidence was correlated with increased dose and young age at the time of exposure. ^25^ Although the risk of dentomaxillofacial imaging using CBCT is small for an individual, when multiplied by the large population of patients exposed to diagnostic imaging, the risk of radiation becomes a significant public health problem, especially in children and adolescents. ^20^

When comparing this study with other bibliometric studies, ^16, 17, 26^ a significant number of self-citations can be observed. Although this practice is not widely accepted in the scientific community, it can be considered a natural consequence. Authors and research groups dedicated to a specific topic may find the need to self-cite their own work in order to contribute to the ongoing development of this area. ^27^

### Year of Publication

The oldest article, published in 2008, proposes the use of reference points for cephalometric analysis using three-dimensional (3D) CBCT reconstruction with orthodontic indications. ^28^ In this study, CBCT scans of 10 adolescents were used, and the authors indicated that the reference point with the highest intra-reliability was located between the two spinous foramina. With the indication of this cephalometric point, the authors proposed a tool that could be used as a reference for orthodontists when analysing 3D images since the use of this type of image, as it is an anatomically true representation (1:1 size), from which the sections can be displayed from any angle in any part of the skull and provided digitally on paper or film, can provide useful information for identifying teeth and other structures for diagnostic and descriptive purposes.

Since 2005, publications investigating or using CBCT in children and adolescents have shown an upward trend over the years. A significant increase in the years 2021, 2022 and 2023 stands out, once again highlighting the relevance of this imaging exam in paediatric dental patients. This trend suggests not only recognition of the clinical importance of CBCT but also reinforces confidence in its safety when applied to this specific age group.

### Publishing Journals

The American Journal of Orthodontics and Dentofacial Orthopedics was the journal with the highest number of published articles and, therefore, the highest number of citations. However, it was well below the JIF, occupying only seventh position. The JIF is the annual average of citations of the articles published by the journal over the last two years. ^29^ It is noteworthy that, despite the International Journal of Paediatric Dentistry and the European Journal of Paediatric Dentistry being the paediatric dentistry journals with the highest JIF, the number of studies published on CBCT (cone beam computed tomography) in these journals remains considerably lower than in the most frequently cited dental journals.

### Study design and themes

The choice of study design plays an important role in the quality and reliability of research findings. In paediatric dentistry, the use of CBCT has been investigated using a variety of study designs, including observational studies, clinical trials, case reports and systematic reviews. Selecting the appropriate study design helps to address specific research questions, such as the applicability of CBCT from clinical diagnosis and treatment planning to the assessment of long-term outcomes.

The predominance of observational studies in this review aligns with previous bibliometric analyses in dental research, where observational designs are often favoured for their ability to collect data in real-world settings. ^11, 12^ While observational studies provide valuable information on trends and associations in clinical practice, their limitations, such as the inability to establish causal relationships and control for confounding variables, should be acknowledged. ^18^ Previous studies, including those on CBCT applications in orthodontics and endodontics, have also highlighted the usefulness of such designs in documenting treatment outcomes and identifying practical applications. ^3, 10^

However, the low proportion of randomised controlled trials (less than 1%, with only five studies) identified in this global review may be attributed to the ethical and practical challenges of conducting trials involving ionising radiation in paediatric populations. Given the risks associated with radiation exposure, particularly the long-term risks of cancer, RCTs in this area are difficult to justify. ^24^ In contrast, controlled studies would offer a more rigorous assessment of outcomes and potential risks, particularly regarding radiation exposure in children. ^6^ Instead, observational studies are more commonly employed, as they allow for the exploration of CBCT’s evolving role in treatments without the need for unnecessary radiation exposure. ^16, 30^

Most of the studies included in this analysis used CBCT to plan and assess maxillary expansion, highlighting the importance of this technology in complex orthodontic diagnoses. CBCT is widely used to evaluate bone morphology and tissue response to rapid maxillary expansion (RME), particularly in children with maxillary atresia. Studies such as Fastuca et al. (2015) ^30^ demonstrate that CBCT provides detailed insight into airway changes following RME, allowing for accurate evaluation of skeletal and dentoalveolar modifications. Additionally, CBCT has proven essential for three-dimensional documentation of bone and dental movement, offering precision in post-treatment follow-up, which facilitates planning for additional interventions. However, the potential for CBCT to reduce radiation exposure in paediatric patients requires further exploration, particularly in optimising image acquisition protocols.

Another significant use of CBCT was in aiding endodontic diagnosis and treatment, with an increasing emphasis on its use to assess root canal volume and untouched areas after instrumentation, as discussed by Gogos et al. (2020). ^11^ CBCT offers high-resolution images, enabling precise three-dimensional visualisation of anatomical structures such as roots and canals, which are often challenging to assess in conventional two-dimensional radiographs. This accuracy facilitates the diagnosis of hidden pathologies and improves the success of endodontic treatments. However, there is an opportunity to expand research on the long-term impact of CBCT on clinical success in endodontic treatments, as well as its application in guided endodontic techniques.

CBCT was also widely used in the diagnosis and treatment of cleft lip and/or palate, where its ability to generate detailed three-dimensional images is indispensable for surgical planning. The clear visualisation of the palate and surrounding areas allows for accurate assessment of the cleft and associated bone structure. The CBCT has been widely used in preoperative planning and postoperative follow-up of these corrections, especially in paediatric patients. However, despite its widespread use, the challenge of minimising radiation exposure in children remains a priority, necessitating further studies focused on developing low-dose imaging protocols. ^16^

### Institutions and Authors

Different institutions carried out most of the studies. The University of Alberta in Canada emerged as the most important institution with the highest number of publications. This prominence can be attributed to the university’s robust research programmes in paediatric dental patients and its significant investment in imaging technologies. Ranked in the top 100 in the world and fourth in Canada, ^31^ the University of Alberta has extensive resources and expertise that are likely to contribute to its high output in this area. In addition, the presence of specialised research centres and strong collaborative networks with other leading institutions may enhance its ability to produce influential research in CBCT and paediatric dental patients, which may explain the significant number of articles in this bibliometric analysis.

Jacobs R was the most prominent author, focusing his articles mainly on observational and laboratory studies. His focus is on the assessment of radiation dose during cone beam computed tomography (CBCT) scans. On the other hand, the second most prominent author is Lagravere MO, whose production covers observational and interventional studies, with a particular emphasis on maxillary expansion. These authors, although clustered in 2018, belong to different research groups without any collaboration. These two authors stand out as significant contributors to the field, each bringing valuable perspectives and knowledge to research related to CBCT and paediatric dental patients.

### Strengths and limitations

The findings of this bibliometric review have important clinical implications for professionals in the field of paediatric dental patients. The principal application of CBCT in maxillary expansion, as evidenced in this review, highlights its efficacy in the differential diagnosis and treatment planning of patients with complex maxillofacial conditions. For clinicians, an awareness of these trends can facilitate the identification of cases in which CBCT should be used to optimise patient outcomes. This encompasses cases that demand precise imaging for orthodontic assessments, surgical planning, or the evaluation of cleft lip and palate. Furthermore, the data suggests an increasing focus on reducing radiation exposure, which is vital for establishing safe practice protocols. Consequently, this review offers clinicians a detailed account of contemporary research trends, thereby facilitating the formulation of optimal practices and providing a foundation for evidence-based clinical decision-making.

Furthermore, this study did not impose any restrictions regarding publication year, citations, or language, thereby enabling a comprehensive and inclusive analysis of all documents pertaining to CBCT in paediatric dental patients up to the date of the research. This appears to be the inaugural bibliometric review to address this topic in such a comprehensive manner.

While this review offers a thorough examination of the use of CBCT in paediatric dental patients based on data from the Web of Science database, it is crucial to recognize certain limitations inherent in this approach. The decision to focus on a single database, excluding others such as Scopus or PubMed, might have restricted the breadth of the analysis. Consequently, some relevant studies published in other databases may not have been considered. This choice aligns with the methodology used in previous bibliometric reviews, ^15, 17^ and was made to maintain consistency with established research practices. Nonetheless, to achieve a more exhaustive understanding of the available literature and reduce the risk of overlooking significant findings, future research would benefit from adopting a more inclusive search strategy that encompasses multiple databases. This approach could help mitigate the potential for biases associated with the selection of a single data source.

## Conclusions

This bibliometric review provided an overview of the current state and trends in research into the use of CBCT for dental purposes in children and adolescents. An important finding was the greater research interest in Asia and Europe. Although the oldest article was published 19 years ago, there has been substantial growth in this area in the last four years, with an emphasis on observational studies aimed at assessing maxillary expansion. It is important to note that most of these studies were published in the American Journal of Orthodontics and Dentofacial Orthopedics, with the United States and China being the most frequent origins of these contributions. This panorama reflects not only the significant advance in research involving CBCT in paediatric dental patients but also points to a geographic distribution that highlights interest and scientific production in this specific area.

## Ethics and consent

Ethical approval and consent were not required.

## Data Availability

Figshare: Dataset Supporting the Bibliometric Review of Cone Beam Computed Tomography in Paediatric Dentistry. https://doi.org/10.6084/m9.figshare.27176001.v5. ^19^ The project contains the following extended data:
• Bibliom_CBCT.xlsx and Data Files CBTC.sav (Raw bibliometric data extracted from the top-cited articles on Cone Beam Computed Tomography (CBCT), supporting the findings of the study), • Flow diagram-v1 Figshare: BIBLIO checklist for ‘A global overview of the use of cone beam computed tomography in dentistry: a bibliometric review focusing on paediatric patients’. pdf. https://doi.org/10.6084/m9.figshare.27176001.v5. ^19^ Figshare: Flow diagram of the selection process of the included studies. https://doi.org/10.6084/m9.figshare.27176001.v5. ^19^ Data are available under the terms of the Creative Commons Zero “No rights reserved” data waiver (CC0 Public domain dedication).
