# Supplementary material for: A global overview of the use of cone beam computed tomography in dentistry: a bibliometric review focusing on paediatric patients
Source: F1000Res. 2025 Apr 10;13:1320. Originally published 2024 Nov 5. [Version 2] doi: 10.12688/f1000research.157349.2 (PMC12009477; doi:10.12688/f1000research.157349.2)
Supplement: Peer review history

# Reviewer report: Reviewer response for version 2

**DOI:** 10.5256/f1000research.178949.r377122

**Published:** 2025-04-19

**Authors:** Beenish Fatima Alam

**Affiliations:** 1 Dentistry, Bahria University, Karachi, Pakistan; 2 Universiti Sains Malaysia, Minden Heights, Pulau Pinang, Malaysia

Author have successfully implemented all the changes as I had suggested.

Is the work clearly and accurately presented and does it cite the current literature?

Yes

If applicable, is the statistical analysis and its interpretation appropriate?

I cannot comment. A qualified statistician is required.

Are all the source data underlying the results available to ensure full reproducibility?

Yes

Is the study design appropriate and is the work technically sound?

Yes

Are the conclusions drawn adequately supported by the results?

Yes

Are sufficient details of methods and analysis provided to allow replication by others?

Yes

Reviewer Expertise:

Bibliometric analysis,

I confirm that I have read this submission and believe that I have an appropriate level of expertise to confirm that it is of an acceptable scientific standard.

# Reviewer report: Reviewer response for version 2

**DOI:** 10.5256/f1000research.178949.r377121

**Published:** 2025-04-18

**Authors:** Çiğdem Şeker

**Affiliations:** 1 Zonguldak Bülent Ecevit University, Zonguldak, Turkey

The revisions made in line with the suggestions are sufficient. It is clear that the study will contribute to its field. I congratulate the authors and wish them success.

Is the work clearly and accurately presented and does it cite the current literature?

Yes

If applicable, is the statistical analysis and its interpretation appropriate?

I cannot comment. A qualified statistician is required.

Are all the source data underlying the results available to ensure full reproducibility?

Yes

Is the study design appropriate and is the work technically sound?

Yes

Are the conclusions drawn adequately supported by the results?

Yes

Are sufficient details of methods and analysis provided to allow replication by others?

Yes

Reviewer Expertise:

Oral diagnosis, Dentomaxillofacial radiology

I confirm that I have read this submission and believe that I have an appropriate level of expertise to confirm that it is of an acceptable scientific standard.

# Reviewer report: Reviewer response for version 1

**DOI:** 10.5256/f1000research.172783.r342266

**Published:** 2025-01-07

**Authors:** Beenish Fatima Alam

**Affiliations:** 1 Dentistry, Bahria University, Karachi, Pakistan; 2 Universiti Sains Malaysia, Minden Heights, Pulau Pinang, Malaysia

Abstract: 

 rephrase this line 'This study provides an overview of CBCT in paediatric dentistry' as This study provides an overview regarding the role of CBCT in paediatric dentistry.

 Methods:

 Timeline for data collections needs to be added.

 Date when the online search was conducted on WOS needs to be added in methods and in abstract. 

 How were papers scrutinized/selected? this processs needs to be described in details. You can add a prisma flow chart to better explain the process of paper selection. 

 inclusion criteria and exclusion criteria need to be clearly described. 

 Discussion:

 'Publishing Journals' more details regarding influence of impact factor of journals and why authors prefer to publish in such journals needs to be discussed.

Is the work clearly and accurately presented and does it cite the current literature?

Yes

If applicable, is the statistical analysis and its interpretation appropriate?

I cannot comment. A qualified statistician is required.

Are all the source data underlying the results available to ensure full reproducibility?

Yes

Is the study design appropriate and is the work technically sound?

Yes

Are the conclusions drawn adequately supported by the results?

Yes

Are sufficient details of methods and analysis provided to allow replication by others?

Yes

Reviewer Expertise:

Bibliometric analysis,

I confirm that I have read this submission and believe that I have an appropriate level of expertise to confirm that it is of an acceptable scientific standard, however I have significant reservations, as outlined above.

# Response

**Published:** 2025-03-03

**Authors:** Danielle Cristina Alves Rigo

**Affiliations:** Odontologia, Universidade Federal de Santa Catarina, Florianópolis, State of Santa Catarina, Brazil

We appreciate the valuable comments and suggestions provided by the reviewers. Below, we address each point and describe the modifications made to the manuscript (v.2) accordingly.

**Response to Reviewer 1**

1. **Metal artifact formation in CBCT**
  Thank you for your observation. We revised the introduction to clarify that CBCT alone does not inherently minimize metal artifacts. Instead, metal artifact reduction (MAR) algorithms and other post-processing techniques play a crucial role in improving image quality. The relevant references have been checked and updated to reflect this correction.
2. **Radiation risks and safety protocols**
  We acknowledge the importance of discussing safety protocols in greater detail. Additional information has been included in the discussion section, emphasizing the importance of ALARA (As Low As Reasonably Achievable), ALADA (As Low As Diagnostically Acceptable), and ALADAIP (As Low As Diagnostically Acceptable for an Individual Patient) principles. We also highlighted recommendations from recent guidelines, such as those from the DIMITRA Project and ICRP Publication 154 (2023), which advocate for individualized dose optimization strategies.
3. **Increase in publications in 2023**
  We have added an explanation regarding the observed increase in publications in 2023. This rise coincides with the release of new recommendations on radiation protection, such as the ICRP Publication 154, which likely stimulated further research in the field. Additionally, technological advancements and increased accessibility to CBCT may have contributed to this trend.

**Response to Reviewer 2**

1. **Abstract revision**
  As suggested, the sentence in the abstract has been rephrased to: "This study provides an overview regarding the role of CBCT in paediatric dentistry."
2. **Timeline for data collection**
  The timeline for data collection has been explicitly stated in the methods section, indicating that the online search was conducted on 22 February 2024. This information has also been included in the abstract for clarity.
3. **Paper selection process**
  Regarding the PRISMA flowchart, we acknowledge the reviewer’s suggestion. However, since this is a bibliometric study following the BIBLIO guideline, a PRISMA diagram was not used. Instead, we adhered to the structured methodology of bibliometric analysis. The rationale for this choice has been explained in the methods section.
4. **Inclusion and exclusion criteria**
  We have revised the description of the inclusion and exclusion criteria to improve clarity and precision. The updated text now explicitly states that only original research articles focusing on CBCT applications in paediatric dentistry were included, while conference papers, editorials, and studies not involving CBCT use in paediatric dental patients were excluded. Additionally, no restrictions on language or publication year were applied to ensure a comprehensive analysis.
5. **Publishing journals and impact factor**
  Additional discussion has been incorporated regarding the influence of journal impact factors and the authors' preference for specific journals. We have clarified that while high-impact journals provide greater visibility, researchers often prioritize subject-specific journals that cater more directly to their audience. This discussion has been expanded in the section on publishing trends.
  We appreciate the reviewers' insightful comments, which have helped improve the clarity and comprehensiveness of our manuscript. We hope that these modifications address all concerns satisfactorily. Thank you for your time and effort in reviewing our work.

# Reviewer report: Reviewer response for version 1

**DOI:** 10.5256/f1000research.172783.r345091

**Published:** 2024-12-18

**Authors:** Çiğdem Şeker

**Affiliations:** 1 Zonguldak Bülent Ecevit University, Zonguldak, Turkey

Visualization of collaboration networks using VOSviewer enhances the study scientifically and visually. The benefits of CBCT in specific areas such as maxillary expansion, endodontic treatment and cleft lip and palate management are well highlighted. The study provides important scientific data by analyzing global trends in the use of CBCT in pediatric dentistry, leading countries, institutions and authors.

 If I may make a suggestion: 1. I could not find any information in the sources cited in the introduction regarding the issue of CBCT minimizing metal artifact formation. On the other hand, this is mostly possible with software programs such as the metal artifact reduction algorithm. I recommend reviewing this information and references.

 2. Radiation risks associated with the use of CBCT in pediatric patients are discussed, but no recommendations for more detailed safety protocols are made. If available in the studies, this topic can also be discussed.

 3. When analyzing the study themes, do you have any idea about the reason for the increase in 2023? If so, please specify.

Is the work clearly and accurately presented and does it cite the current literature?

Yes

If applicable, is the statistical analysis and its interpretation appropriate?

I cannot comment. A qualified statistician is required.

Are all the source data underlying the results available to ensure full reproducibility?

Yes

Is the study design appropriate and is the work technically sound?

Yes

Are the conclusions drawn adequately supported by the results?

Yes

Are sufficient details of methods and analysis provided to allow replication by others?

Yes

Reviewer Expertise:

Oral diagnosis, Dentomaxillofacial radiology

I confirm that I have read this submission and believe that I have an appropriate level of expertise to confirm that it is of an acceptable scientific standard, however I have significant reservations, as outlined above.
